# Association of the clinical profile and overall survival of pediatric patients with acute lymphoblastic leukemia

**DOI:** 10.3389/fped.2023.1223889

**Published:** 2023-09-07

**Authors:** Bruno Terra Correa, Gabriela Sales Serra Silva, Webert Joaquim Silva Mendes, Amanda de Moraes Maia, Augusto Cezar Magalhães Aleluia, Teresa Cristina Cardoso Fonseca, Caroline Conceição da Guarda, Marilda de Souza Gonçalves, Milena Magalhães Aleluia

**Affiliations:** ^1^ Departamento de Ciências Biológicas, Laboratório de Patologia Aplicada e Genética, Universidade Estadual de Santa Cruz, Ilhéus, Bahia, Brasil; ^2^ Departamento de Medicina, UNEX, Itabuna, Bahia, Brasil; ^3^ Departamento de Ciências Naturais, Universidade Estadual do Sudoeste da Bahia-Campus de Vitória da Conquista, Vitória da Conquista, Bahia, Brasil; ^4^ Departamento de Ciências da Saúde, Universidade Estadual de Santa Cruz, Ilhéus, Bahia, Brasil; ^5^ Laboratório de Investigação Genética e Hematologia Translacional, Instituto Gonçalo Moniz, Fundação Oswaldo Cruz, Salvador, Bahia, Brasil

**Keywords:** acute lymphoid leukemia, survival, pediatric cancer, adverse effects, chemotherapy

## Abstract

**Introduction:**

The clarification of etiopathology, the improvement of chemotherapy regimens and their risk stratifications, and the improvement in treatment support have increased the survival of children and adolescents affected by Acute Lymphoblastic Leukemia (ALL) past few years. This study aimed to estimate overall survival (OS) and event-free survival (EFS) in an onco-hematology treatment center in Brazil, reports the main clinical-laboratory characteristics of patients at diagnosis, verify the frequency of treatment-related adverse effects and the main causes of death.

**Material and methods:**

Retrospective analysis involving patients diagnosed with ALL, treated with the protocol of the Brazilian Group for Treatment of Leukemias in Childhood (GBTLI), between 2010 and 2020 was carried out; the outcomes (relapse, deaths, development of new neoplasms) were analyzed SPSS® software was used for the statistical analyses, and the *p*-value was considered significant when less than 0.05 for all analyses.

**Results:**

109 patients were included in the study; the median age was 5 years, with a slight predominance of males. Sixty-six patients were classified as high-risk (HR) group and 43 patients were classified as low-risk (LR) group. After 5 years of diagnosis, the OS was 71.5%, and the EFS was 65%. No statistical difference was found between the HR and LR groups for OS and EFS, while leukocyte counts were statistically associated with the outcome of death (*p *= 0.028). Among the patients, 28 (25.6%) died due to infection accounting 46.4% of death causes. Among the 34 patients with unfavorable outcomes (death and/or relapse), 32 had no research for the minimal residual disease at the end of remission induction, and 25 were not investigated for the presence of chromosomal abnormalities. The most reported complications and treatment-related adverse effects were increased liver transaminases (85.9%), airway infection (79.4%), oral mucositis (67.2%), febrile neutropenia (64.4%), and diarrhea (36.4%).

**Conclusions:**

The rates of OS and EFS obtained in this cohort are similar to those obtained in the few previous similar studies in Brazil and lower than those carried out in developed countries. The unavailability of prognostic tests may have hindered risk stratification and influenced the results obtained.

## Introduction

Acute Lymphoblastic Leukemia (ALL) is the most common type of cancer affecting the pediatric population, with a higher incidence between 0 and 4 years of age (1). In the United States, about 3,000 patients aged 1–19 years are diagnosed with this pathology yearly (2). First-line treatment for ALL consists of a combination regimen of chemotherapeutic agents, which usually includes the phases of remission induction, consolidation, intensification, and maintenance. Allogeneic hematopoietic stem cell transplantation is restricted to patients at very high risk of relapse, those with persistent minimal residual disease (MRD) throughout treatment, and cases of relapse (3).

In recent decades, amendments in chemotherapy treatment protocols and improved supportive care have made it possible to increase survival in children and adolescents with ALL. Progress in the etiopathological understanding of the disease has also been aiding in the progression of outcomes, as it enables better risk stratification and the development of targeted therapies (3–5). Survival rates for children aged 0–14 years before 1990 were around 73%, and after 2010, there was a substantial increase reaching up to 93% (6). However, these outcome improvements occur primarily in developed countries (7). In developing countries, survival rates are usually lower, even though chemotherapy treatment protocols are similar (8). It is plausible that this inferiority in outcomes results from the shortage of high-cost diagnostic and prognostic tests, with the consequent difficulty of proper and individualized diagnosis and correct risk stratification (9). It is also likely that inadequate hospital support infrastructure and demanding access to specialized treatment centers may contribute to unfavorable outcomes (8). Many clinical characteristics may modulate the outcome of patients with ALL. Based on the immunophenotype, patients with T-cell ALL have worse outcomes than patients with B-cell ALL. T-cell ALL is also more frequent among male patients; some genetic markers are related, although the precise mechanism is not fully understood. In addition, adolescents (10–19 years old) present more treatment-related morbidities (10).

Cancer epidemiology and survival rates data are poorly reported in developing countries (11). According to data from the Instituto Nacional de Câncer (INCA), the estimated risk of childhood cancer (aged 0–19 years) for 2023–2025 in Brazil is 135 per million. Regarding the distribution by sex, the risk is estimated at 140 per million for males and 129 per million for females. A similar value is estimated for the world population (155.8 per million) (12). The incidence, prevalence, mortality, and survival rates are essential health system quality indicators and are able to elucidate possible barriers related to unfavorable outcomes, as well as potential solutions for improving healthcare (13).

Considering the lack of data in the literature regarding pediatric ALL in Brazil, its low survival rates compared to developed countries, and its clinical relevance, it is necessary to investigate the pediatric population affected by ALL and identify the factors associated with unfavorable outcomes. Thus, the objective of this study was to analyze the treatment outcomes of ALL in children and adolescents as well as the clinical-epidemiological profile in an onco-hematology treatment center.

## Material and methods

### Patients and study design

We developed an analytical observational study of a retrospective cohort type, analyzing clinical, laboratory, and outcome data of patients aged up to 19 years, diagnosed with ALL and treated at Unidade de Alta Complexidade em Oncologia (UNACON) of Santa Casa de Misericórdia de Itabuna (Bahia—Brazil), between 2010 and 2020. Clinical and laboratory data were obtained from physical and electronic medical records. This study received approval from the ethics and research committee of Universidade Estadual de Santa Cruz (UESC) through protocol N°. 47456221.0.0000.5526 and was carried out in accordance with the ethical principles established by the Helsinki Declaration (1964) and its subsequent amendments and by resolution number 466 of the Brazilian National Health Council of December 12, 2012.

### Diagnosis of ALL

The criterion used for diagnosis of ALL in the study center was the presence of equal to or greater than 25% of lymphoblasts in the bone marrow (BM). In cases where the bone marrow aspirates samples were inadequate for analysis, an osteomedullary biopsy was performed for diagnostic confirmation. The presence of 5 blasts/mm^3^ or more in the cerebrospinal fluid (CSF) was indicative of central nervous system involvement by ALL. Flow cytometry immunophenotyping, cytogenetic study, and molecular analysis were performed.

### Treatment protocol

**Figure 1:**
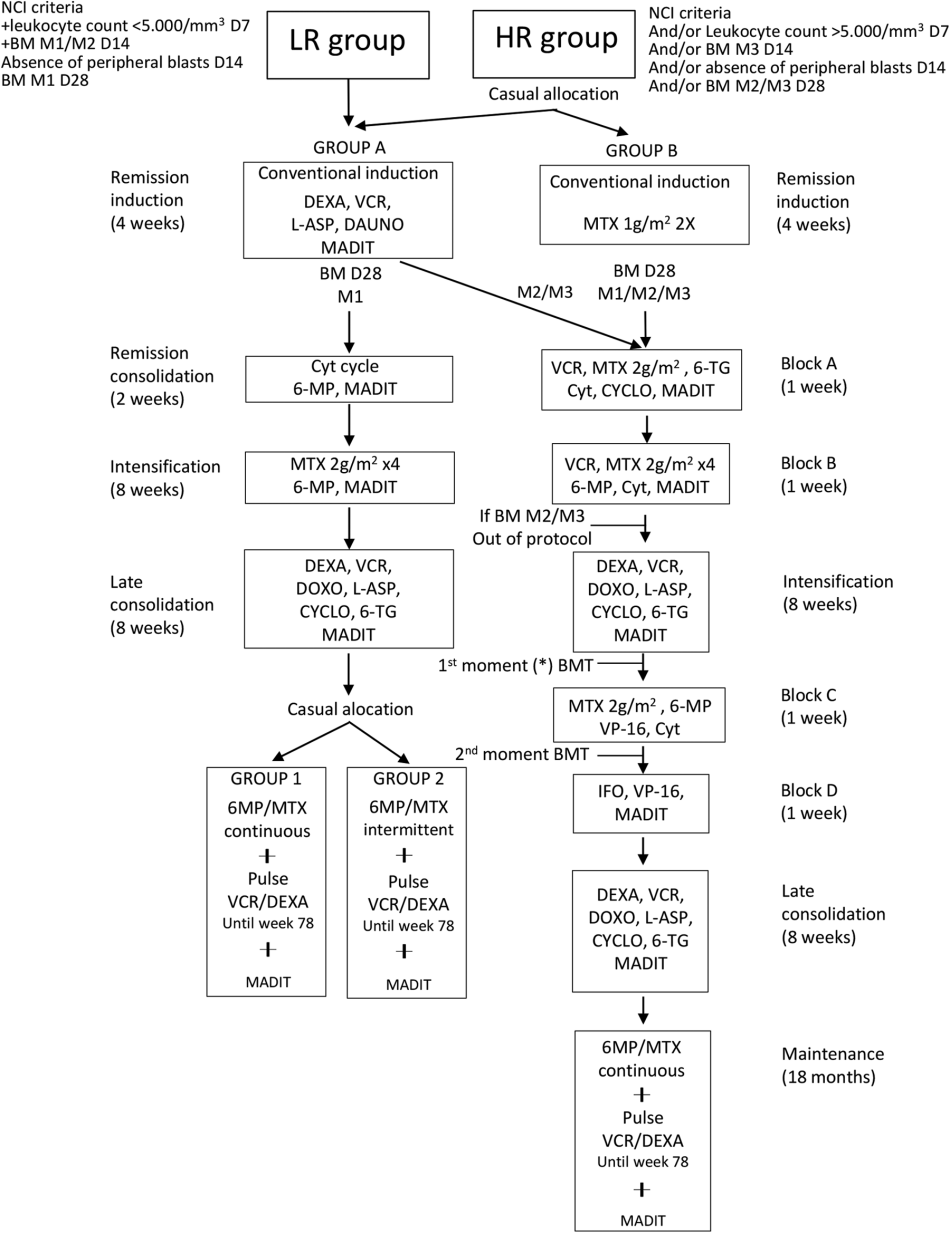
General scheme of the GBTLI-99 protocol. NCI, *national cancer institute*; BM, bone marrow; M1, bone marrow with <5% of lymphoblasts; M2, bone marrow with 5 to 25% of lymphoblasts; M3, bone marrow with 25% of lymphoblasts; D7, D14, D28, treatment days; LR, low risk; HR, high risk; without—weeks; DEXA, dexamethasone; VCR, vincristine; L-ASP, asparaginase; DAUNO, daunorubicin; MADIT, methotrexate + cytarabine + dexamethasone intrathecal; MTX, methotrexate; Cyt, cytarabine; 6-MP, mercaptopurine; 6-TG, thioguanine; CYCLO, cyclophosphamide; DOXO, doxorubicin; VP16, etoposide; IFO, ifosfamide; BMT, bone marrow transplantation; *allogeneic bone marrow transplantation indicated in the group of slow responders (presence of peripheral blasts and/or M3 marrow on D14) that present unfavorable cytogenetic alteration at diagnosis [eg Ph or t (4;11)]. Patients with inductive failure will also be included. Adapted from the original GBTLI-99 protocol of the study treatment center (13).

The protocol of the Brazilian Group for the Treatment of Leukemias in Childhood 99 (GBTLI-99) (14) stratifies the patients into two main groups: Low Risk (LR) and High Risk (HR). The inclusion criteria for the HR group were: age <1 year or age ≥10 years or older, and/or leukocyte counts at diagnosis ≥50,000/mm^3^, and/or slow response rate to the treatment (leukocyte counts ≥5,000/mm^3^ on D7, presence of peripheral blood lymphoblasts or marrow lymphoblasts >25% on D14), and/or non-responsive patients (marrow lymphoblasts >5% on D28). Other criteria for inclusion in the HR group were evidence of extramedullary leukemic involvement at the end of induction and the presence of lymphoblasts in the cerebrospinal fluid (CSF) on D14. In the HR group, Allogeneic Hematopoietic Stem Cell Transplantation could be indicated among the poor responders (presence of peripheral or even medullary blasts (if above 25%) on day 14 of induction, and with cytogenetic findings of poor prognoses, such as t(9;22) or t(4;11). Those with remission induction failure would also have this therapeutic indication. The general scheme of the protocol is depicted in Figure 1.

Patients with mature B-cell ALL and those who discontinued the treatment at the study center due to transfer to other centers were excluded from the study.

### Definition of adverse effects

Adverse effects and complications secondary to treatment were those events reported after the start of therapy, with no apparent leukemic etiology. The results of laboratory and imaging tests were considered, as well as reports in medical records of signs and symptoms suggestive of adverse events.

### Statistical analysis

The data obtained from the medical records were tabulated and analyzed using SPSS® software version 20.0 (IBM Software, New York, USA). The descriptive analyses of the clinical characteristics of the study participants were expressed as means and standard deviations or median and interquartile range (IQR). Associations between variables, prognostic factors, and responses were analyzed according to the chi-square, Fisher or Mann–Whitney tests. Multivariate logistic regression was also performed to analyze the association between clinical characteristics and outcomes, and the covariates were selected based on published clinical criteria.

Overall survival (OS) was estimated from the date of diagnosis until the date of death or the date of the last visit. Event-free survival (EFS) was estimated from the date of diagnosis until the date of the first event. Relapse, death, or a new malignant neoplasm diagnosis were considered events. OS and EFS were calculated according to Kaplan–Meier survival analysis, and the log-rank test compared the curves. The *p*-value was considered significant when less than 0.05 for all analyses.

## Results

### General characteristics of patients with ALL

Initially, 111 patients were selected for the study, but 2 were excluded due to transfer for treatment at other centers. Therefore, 109 patients were included in the present study. The patients were from 47 different cities that were related to the onco-hematology center. The clinical and laboratory characteristics at diagnosis are summarized in Table 1.

**Table 1:** Clinical and laboratory characteristics of patients with ALL at diagnosis.

Characteristics at diagnosis	*N* (%)
Sex	
Female	47 (43.1)
Male	62 (56.9)
Age	
Median/IQR	5/3–10
Variação	0,6–18
Mean ± SD	6.5 ± 4.4
Age group (years)	
< 1	80 (73.3)
≥ 10	29 (26.6)
Leukocyte counts (por mm^3^)	
0 to 50,000	81 (74.4)
50,001 to 100,000	14 (12.8)
> 100,000	14 (12.8)
Immunophenotype	
Pre-pre-B	3 (2.7)
Pre-B CD10^+^	80 (73.3)
Pre-B	8 (7.3)
T	9 (8.2)
NA	9 (8.2)
Central nervous system impairment	
Positive	4 (3.6)
Negative	105 (96.4)
Chromosomal abnormalities	
t(12;21)	7 (6.4)
t(1;19)	1 (0.9)
t(9;22)	1 (0.9)
t(4;11)	0
t(11q23)	1 (0.9)
Hyperdiploidy	1 (0.9)
Hypodiploidy	0
Normal karyotype	3 (2.7)
Complex karyotype	0
No mitosis	2 (1.8)
NA	71 (65.1)
Risk group	
High risk	66 (60.5)
Low risk	43 (39.5)
Minimal residual disease at the end of induction	
Positive	6 (5.5)
Negative	11 (10)
NA	92 (84.4)

IQR, interquartile range; SD, standard deviation; NA, not available, mm^3^, cubic millimeter; ALL, acute lymphoblastic leukemia.

The median age of the patients was 5 years, ranging from 6 months to 18 years. There was a slight predominance of males (*n* = 62, 56.9%). The median time between the onset of the first symptoms and diagnosis was 29 days. Late diagnosis, over 30 days, was not associated with higher frequency of deaths (*χ*² = 2.74, *p *= 0.07).

Regarding the treatment protocol, 88.9% of patients were treated with GBTLI-99 and 10.2% with GBTLI-93. Only 1 patient, who was a carrier of t(9;22), was treated with GBTLI-99Ph. Of 109 patients, 66 (60.5%) were included in the HR group, and 43 (39.5%) in the LR group. A higher frequency of male patients was observed in the HR group compared to female patients (43 and 19 patients, respectively; *p *= 0.047). Moreover, we found that HR patients presented a median value of 30 days (Interquartile Range: 15–60 days) from onset of the symptoms until diagnosis, while LR patients had a median value of 15 days (IQR: 13–30 days) (*p*-value = 0.003). In our cohort of patients, the laboratory exams designed to investigate chromosomal abnormalities and research of MRD were not performed in 71 individuals (65.1%) and in 92 patients (84.4%), respectively.

### Frequency of relapse and death among patients with ALL

Thirty relapses were reported, 7 were early relapses (i.e., detected less than 18 months after diagnosis), and of these, 6 were identified in the HR group. All patients who relapsed early progressed to death. The characteristics of the patients who relapsed can be seen in Table 2. The most frequent site of relapse was the BM (28 patients). Exclusive relapse in the central nervous system (CNS) occurred in only 2 patients.

**Table 2:** Characteristics of patients who relapsed and/or died.

Characteristics at diagnosis	Deaths *N* = 28 (%)	Relapses *N* = 30 (%)
Sex		
Female	10 (35.7)	11 (36.7)
Male	18 (64.3)	19 (63.3)
Age at diagnosis (years)		
Median	5	5
Age groups (years)		
< 1	19 (67.8)	21 (70)
≥ 10	9 (32.1)	9 (30)
Leukocyte counts (por mm^3^)		
0 to 50,000	19 (67.8)	21 (70)
50,001 to 100,000	2 (7.1)	2 (6.7)
> 100,000	7 (25)	7 (23.3)
Immunophenotype		
Pre-pre-B	1 (3.6)	1 (3.3)
Pre-B CD10^+^	23 (82.1)	22 (73.3)
Pre-B	–	2 (6.7)
T	2 (7.1)	3 (10)
LLA without immunophenotype	2 (7.1)	2 (6.7)
Risk group		
High risk	16 (57.1)	18 (60)
Low risk	12 (42.8)	12 (40)

mm^3^, cubic millimeter; ALL, acute lymphoblastic leukemia.

Among the 109 patients, 28 died (25.6%). Among these 28 patients, 24 had a previous diagnosis of relapse, and 4 patients were on their first treatment (3 during remission induction and 1 during maintenance). Infectious conditions were reported as the leading cause of death in 13 patients (46.4%). Other causes reported were hemorrhage, progression of the underlying disease, and acute renal failure.

A multivariate logistic regression model was used to test the association between clinical and laboratory characteristics and patient outcome, with the clinical outcome (life or death) as the dependent variable. Thus, leukocyte counts, gender, risk stratification, immunophenotyping, and minimal residual disease on D30 were dependently associated with the clinical outcome (alive or dead) (R^2^ = 0.097; *p *< 0.0001). In this model, we suggest that leukocyte counts were independently associated with clinical outcomes in patients with ALL (*p *= 0.028). The GBTLI93 protocol did not measured minimal residual disease on D30 (Table 3).

**Table 3:** Binary logistic regression model with clinical variables of ALL in association with patient outcome.

Independent variables	Dependent variable	*p*-value	Exp (B)/OR	IC 95%	β	R^2^	*p*-value
Leukocyte counts		**0** **.** **028**	2.187	1.090–4.389	0.783		
Sex		0.251	0.575	0.224–1.479	−0.553		
Risk stratification	Alive or dead	0.204	1.425	0.825–2.462	0.354	**0** **.** **097**	**0** **.** **000**
Immunophenotype		0.377	0.854	0.601–1.213	−0.158		
Minimal residual disease 30		0.236	1.830	0.673–4.975	0.604		

ALL, acute lymphoblastic leukemia; OR, odds ratio; IC, confidence interval. Bold values indicate significance at *p* < 0.05.

### Overall survival and event-free survival

**Figure 2:**
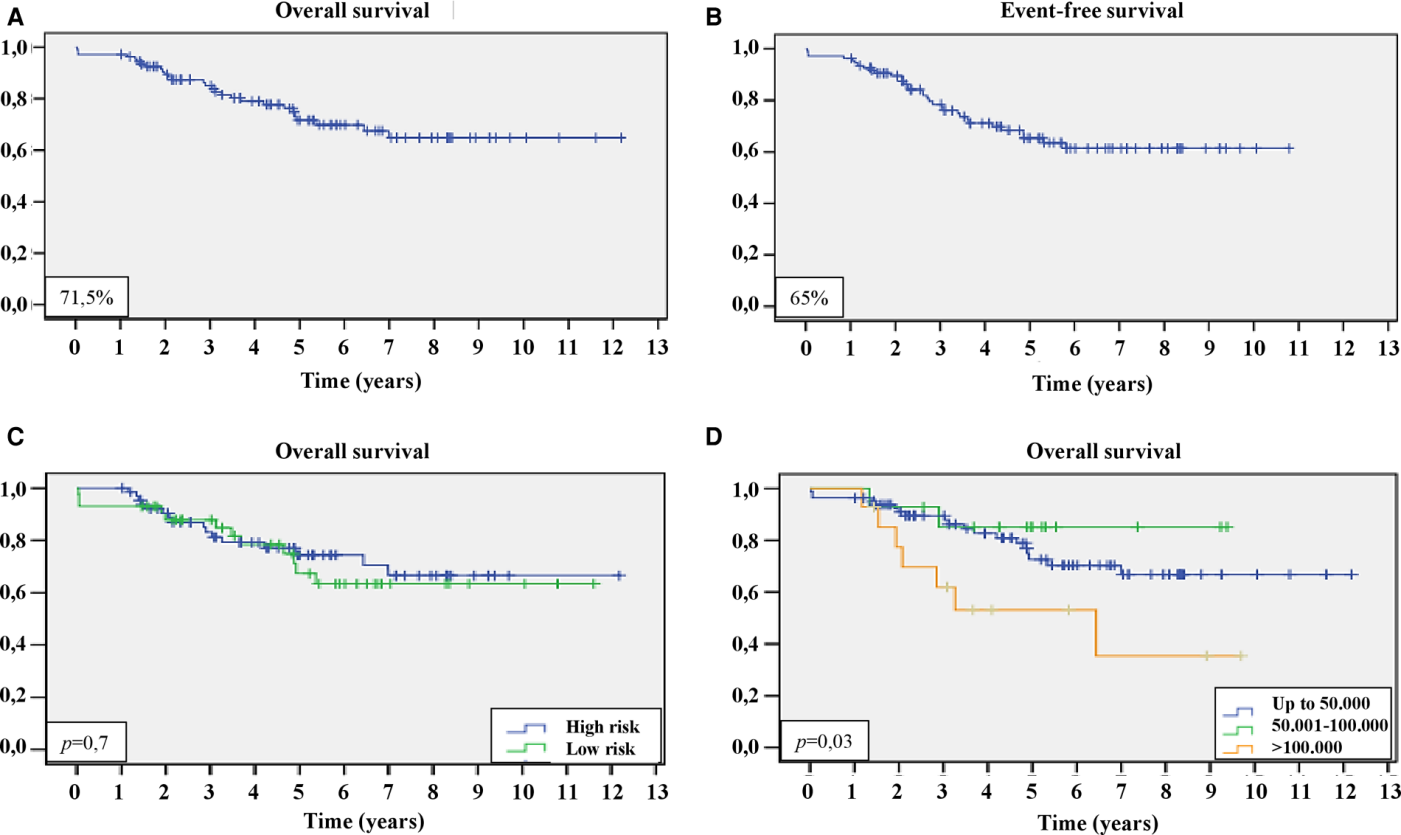
Survival curves of the patients with ALL included in the present study. (**A**) Kaplan–Meier curve of overall survival; (**B**) Kaplan–Meier curve of event-free survival; (**C**) Kaplan–Meier curve of overall survival, comparing patients at high-risk (blue) and low-risk (green) groups; and (**D**) Kaplan–Meier curve of overall survival, comparing patients with leukocyte counts below 50,000/mm^3^ (blue), from 50,001/mm^3^ to 100,000/mm^3^ (green), and above 100,000/mm^3^ (orange), with comparative statistical analysis by *log-rank*. For these analyses *p* < 0.05 was considered statistically significant.

After 5 years of diagnosis, the estimated OS was 71.5%, while the estimated EFS was 65% (Figure 2). There was no statistical difference in OS and EFS between the HR and LR groups, nor was there a statistical difference in OS and EFS between the sexes.

Leukocyte counts higher than 100,000/mm^3^ were associated with OS inferiority (*p *= 0.03). Only 1 patient was diagnosed with a new neoplasm (Acute Myeloid Leukemia) during treatment.

### Frequency of adverse events caused by the treatment

**Figure 3:**
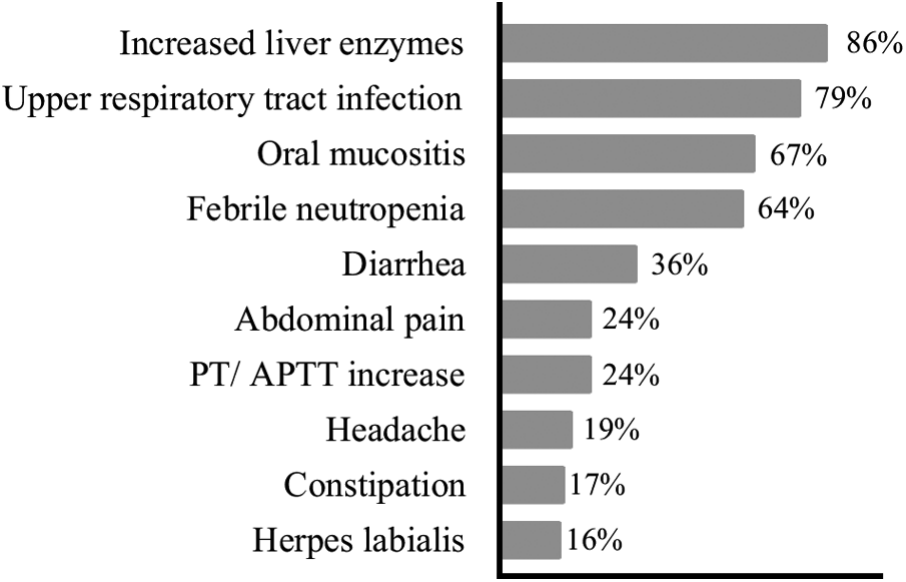
Percentage frequency of adverse events and clinical complications presented by the patients with ALL during the treatment period. PT, prothrombin time; APTT, activated partial thromboplastin time; ALL, acute lymphoblastic leukemia.

Among the adverse effects and complications secondary to treatment, increased liver transaminases were observed in 85.9% of patients, predominantly during the maintenance phase. Upper airway infections were found in 79.4% of patients. Episodes of febrile neutropenia were observed in 64.4% of patients, which were higher in the HR group (*p *< 0.037) compared to the LR group. Figure 3 shows the most frequently reported infectious and non-infectious adverse effects.

## Discussion

The therapeutic protocols for ALL have evolved over the last years with diagnostic techniques in molecular biology and cytogenetics, which allowed better risk stratification and, consequently, a substantial increase in the OS and EFS of patients. In Brazil, few single-center, retrospective cohort-type studies present literature data about pediatric ALL survival (11, 15–21). The OS rates obtained by such studies vary from 29 to 76.5%, consistently lower than those observed in developed countries, as previously shown in studies developed in the Netherlands with OS of 91% (22), in the United Kingdom with OS of 89% (23), and in the United States with OS of 94% (24). The survival results obtained in our study, which pioneered the analysis of pediatric patients with ALL in southern Bahia, Brazil, are in agreement with those obtained in previous national studies, as well as with results from other developing countries (25–29). The global disparity regarding survival in pediatric ALL is evident, even though chemotherapy regimens are very similar (8, 9) between developed and emerging countries.

Proper identification of LR patients may improve OS during pediatric ALL treatment in developing countries, which typically account for more than 50% of cases. These patients may receive lower-intensity chemotherapy treatment, increase the odds of cure, and would be less exposed to treatment-related adverse effects (9). Among the Brazilian studies, the exception is the Rella05 study protocol, conducted in Recife, Brazil, which proposed systematically performing the MRD research by flow cytometry at the end of remission induction. With this prognostic tool, it was possible to properly allocate many patients to the LR group when the MRD research was negative. Thus, these patients received less intense chemotherapy treatment, reducing adverse effects and treatment-related fatal complications. Therefore, an OS of 96% was achieved in this group (30), matching the results expected for developed countries.

Relapse is thought to be related to decreased survival. Thus, many patients who relapsed end up dying due to the toxicity of the restart of chemotherapy as well as the toxicities related to Allogeneic Hematopoietic Stem Cell Transplantation (31). The findings of our study support these data since 24 deaths occurred among the 30 relapses. Therefore, when there is not enough clinical-laboratory evidence for patient allocation to HR or LR group, allocation as HR is often used to reduce the chance of relapse upon receiving more intense treatment (9). However, the higher intensity of chemotherapy treatment can, in turn, add more severe adverse effects, which can be fatal, especially in developing countries that generally have inadequate hospital infrastructure to address such events (30).

This study allocated 66 (60.5%) patients to the HR group. Of these, 40 did not have access to cytogenetic and molecular studies at diagnosis and the possibility of evaluating MRD at the end of induction to determine rapid response to treatment, characterizing insufficient data for proper risk stratification. Six patients were allocated to the HR group due to the use of corticoids prior to diagnosis before entering the treatment center. The use of corticosteroids before treatment can cause rapid destruction of lymphoblasts (32) and, consequently, a decrease in leukocyte counts, thus interfering with the prognostic value of the latter at diagnosis.

Our study evidenced a predominance of males in the HR group with statistical relevance, in contrast to two recent national studies (18, 30). However, no statistically significant difference between OS and EFS between the sexes was evidenced in this study. In our cohort, diagnosis >30 days was associated with risk stratification. The same period was observed in a different study, which reported 30 days from the onset of the symptoms to the first hospital visit (33). In terms of the frequency of the deaths, no statistically significant association was found. Nevertheless, a recent study suggests that patients whose treatment begins within 30 days had a statistically improved survival rate (33).

Furthermore, among the 34 patients with unfavorable outcomes (relapse and/or death), 32 did not have MRD testing at the end of remission induction, and 25 did not have any chromosomal alteration testing (G-banding karyotype, FISH, or molecular biology methods). These tests are not available at the study treatment center and require costly and complex logistics of air shipment of biological material for analysis at the specialized laboratory of the Instituto Nacional de Câncer, in Rio de Janeiro state, Brazil. This center is a contracted center for GBTLI diagnostics. In Brazil, few centers perform these exams, so the material aspirated from the BM at diagnosis must be sent for analysis (34). Moreover, even in these reference laboratories, sometimes there is a lack of materials to perform these exams (35, 36).

The unavailability of prognostic tests mentioned above, as is the case of research on MRD, cytogenetics, and molecular analysis, hindered the proper risk stratification, which may have led more patients to the allocation to the HR group, with the consequent toxicities associated with higher intensity of treatment. The unavailability of these tests may also have been a critical factor for the possible inadequate allocation of patients in the LR group, since a high relapse rate was verified in this group, and, therefore, no statistical difference in OS and EFS was seen between the HR and LR groups.

Elevated leukocyte counts are a well-established negative prognostic factor (3). In this study, leukocyte counts greater than 50,000/mm^3^ (but less than 100,000/mm^3^) were not associated with worse outcomes. On the other hand, leukocyte counts equal to or higher than 100,000/mm^3^ were associated with worse overall survival (*p* = 0.03), as observed in another national study (18).

In this study, the logistic regression model suggests that clinical characteristics (leukocyte counts, gender, risk stratification, immunophenotyping, and minimal residual disease) are dependently associated with the clinical outcome of patients (alive or dead), and leukocyte counts were independently associated with the outcome, corroborating data in the literature (37–39).

Another factor to be considered is the need for better support in treatment backup hospitals. Chemotherapy's complications and adverse effects can lead to fatal outcomes when not adequately addressed. In developing countries, due to financial shortages, some of the support hospitals do not have the adequate infrastructure (40). In specialized centers, oncological and supportive diagnosis and treatment can increase survival chances (41).

Febrile neutropenia, found with great frequency in this study, also requires treatment by trained personnel in the backup hospital. This event is an emergency that can lead to death when not treated with intravenous broad-spectrum antibiotics. Like other types of infection, febrile neutropenia occurs more frequently in the HR patients group (as we found herein), due to the greater intensity of chemotherapy treatment with consequent bone marrow toxicity (42, 43). Infections were also the most significant cause of death (46.4%) in this study, corroborating findings from previous studies (31, 43).

In places with few onco-hematology treatment centers, such as developing countries, the distance between the patient's home and the treatment center may hinder treatment success. Some studies suggest that excessive distance can lead to reduced attendance at consultations, the difficulty of access in the face of intercurrence related to the disease or treatment, and even the abandonment of treatment by the difficulty of locomotion (44, 45). The treatment center of our study is a reference for pediatric onco-hematology treatment in the region and has pacts with several surrounding municipalities. For those living farther away, a vacancy is available at the support house belonging to the center, maintained by the philanthropic institution Grupo de Apoio à Criança com Câncer (GACC). This availability has made it possible for many to continue the treatment in the early stages of the protocol when visits to the treatment center are more frequent. Thus, it was possible to reduce patients' lack of adherence and therapy abandonment from distant cities.

Prospective studies, combined with data already obtained in national studies, may elucidate the reality of pediatric ALL in Brazil, enabling public policies directed towards more promising results. In this sense, a study with the new GBTLI protocol (GBTLI-21) proposed by the Sociedade Brasileira de Oncologia Pediátrica (SOBOPE) is underway. With financial support from the Confederação Nacional de Instituições de Suporte e Assistência às Crainças e Adolescentes com Câncer (Coniac) and with an international partnership with St Jude Children's Research Hospital and the Keira Grace Foundation, there will be prospective data collection from pediatric ALL patients who join this protocol. This prospective research has the perspective of tracing a more reliable profile of children and adolescents with ALL in Brazil and may point to directions for results more comparable to those of developed countries (46).

## Conclusion

Our single-center experience found that 5-year OS was 71.5%, and the estimated EFS was 65%. Statistical analysis revealed no significant difference in OS or EFS between the HR and LR groups; infectious conditions were the primary cause of death. In our cohort, the rates of OS and EFS are comparable to those reported in a few prior similar studies in Brazil though lower than those conducted in developed countries. The absence of prognostic tests may have interfered with risk stratification, which may have impacted the seen outcomes.

## Data Availability

The raw data supporting the conclusions of this article will be made available by the authors, without undue reservation.
